# Strong circularly polarized luminescence from the supramolecular gels of an achiral gelator: tunable intensity and handedness†
†Electronic supplementary information (ESI) available: Additional figures. See DOI: 10.1039/c5sc01056j



**DOI:** 10.1039/c5sc01056j

**Published:** 2015-04-30

**Authors:** Zhaocun Shen, Tianyu Wang, Lin Shi, Zhiyong Tang, Minghua Liu

**Affiliations:** a Beijing National Laboratory for Molecular Science , CAS Key Laboratory of Colloid , Interface and Chemical Thermodynamics , Institute of Chemistry , Chinese Academy of Sciences , Beijing 100190 , P. R. China . Email: liumh@iccas.ac.cn ; Email: twang@iccas.ac.cn ; Tel: +86-10-8261-5803; b Laboratory of Nanomaterials , National Center for Nanoscience and Technology , Beijing 100190 , P. R. China

## Abstract

Although the importance of circularly polarized luminescence (CPL) materials has been widely recognized, the CPL responses of supramolecular gels are still rarely studied. Moreover, developing CPL materials based on supramolecular gels is of great significance, due to their special advantages and important applications. Herein, we report the first circularly polarized supramolecular gels self-assembled exclusively from a simple achiral *C*_3_-symmetric molecule. Most importantly, the excellent tunability of these novel CPL materials, which benefits from achiral molecular building blocks as well as the nature of supramolecular gels, has been investigated. Thus, the CPL intensity of these supramolecular gels is easily enhanced by mechanical stirring or doping chiral amines. The handedness of CPL signals is controlled by the chirality of organic amines.

## Abstract 2

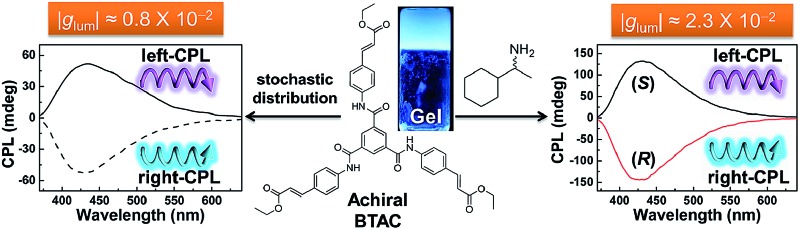
Supramolecular gels formed by an achiral gelator emit strong circularly polarized luminescence with tunable intensity and handedness.

## Introduction

Recently, developing materials exhibiting circularly polarized luminescence (CPL) has attracted more and more interest,^1^ not only for understanding the mysteries of chirality,^2^ but also due to the important potential applications of CPL materials. Chiral molecular systems showing differential emission of right-handed and left-handed circularly polarized light have been explored to be used as display devices,^3^ optical storage devices,^4^ CPL sensors,^5^ biological probes^6^ and even catalysts for asymmetric photochemical synthesis.^7^ Although various chiral molecular systems, such as chiral metal complexes,^1h,8^ chiral polymers,^9^ liquid crystals^1e,10^ and a few chiral organic molecules in solution^1b,11^ or in the solid state,^12^ have been found to be CPL candidates, the supramolecular gels with the ability to emit circularly polarized light are still rarely constructed. Actually, supramolecular gels based on the self-assembly of small organic molecules are very important soft materials with many exceptional advantages, such as flexibility, biocompatibility and easy processing.^13^ Therefore, for the application of CPL systems, supramolecular gels emitting circularly polarized light could be of special significance.

In addition, the known organic molecules showing CPL are usually chiral molecules with complicated but specific structures, for example chiral helicenes,^1h,12b,14^ which sometimes need tedious synthetic processes. Therefore, developing new molecules with more simple structures and the capability of emitting strong CPL signals becomes extremely important for constructing novel CPL devices.

**Fig. 1:**
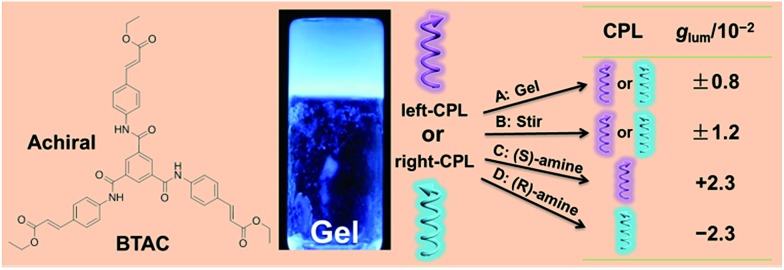
First circularly polarized supramolecular gels self-assembled exclusively from a simple achiral *C*_3_-symmetric molecule (BTAC). The amplitude of CPL signals can be further enhanced upon stirring or adding chiral amines, and the handedness of CPL signals can be controlled well by the chirality of organic amines.

We also notice that almost all of the CPL materials are based on chiral molecular systems. The only known exception from the literature is the co-assembly of an achiral ionic polymer and Rhodamine B, which shows CPL under mechanical stirring.^15^ Our previous study has demonstrated that simple achiral *C*_3_-symmetric molecules (tris(ethyl cinnamate) benzene-1,3,5-tricarboxamides, BTAC) can self-assemble into supramolecular gels with intense circular dichroism (CD) signals (Fig. 1).^16^ In this paper, we present supramolecular gels that show excellent CPL performance (luminescence dissymmetry factor, *g*_lum_ = ±0.8 × 10^–2^), even though these gels are composed of identical achiral organic molecules. Most importantly, both the intensity and handedness of CPL signals of these gels can be easily modulated. Upon mechanical stirring, the CPL intensity can be enhanced; while adding some simple chiral dopants not only can enhance the amplitude of CPL signals but also can readily control the handedness of CPL signals (Fig. 1).

## Results and discussion

### CPL activity of BTAC gels

**Fig. 2:**
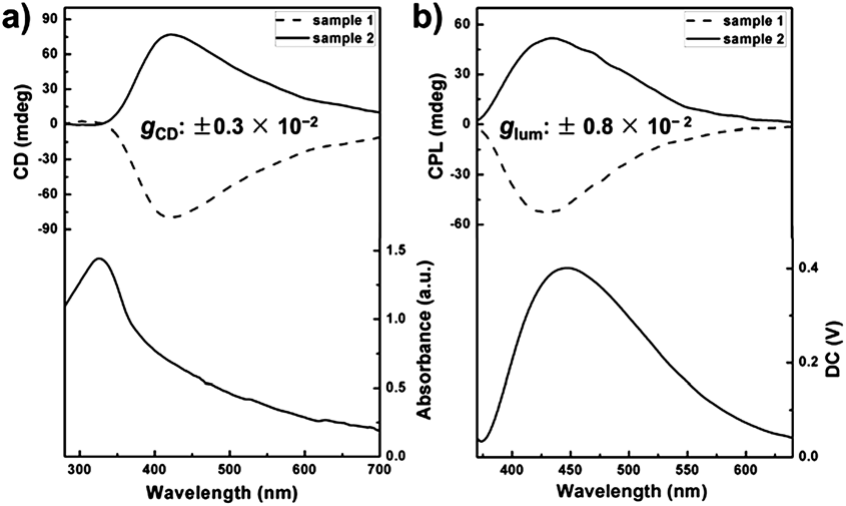
(a) CD (left axis) and UV-Vis (right axis) spectra of BTAC gels (1% w/v) in DMF/H_2_O (v/v: 5/2); (b) CPL spectra (left axis) and fluorescence spectra (right axis) of BTAC gels (1% w/v) in DMF/H_2_O (v/v: 5/2) excited at 330 nm. The sample with negative Cotton effect displays right-handed CPL (dash curves), while the sample with positive Cotton effect exhibits left-handed CPL (solid curves).

BTAC is a *C*_3_-symmetric molecule with three aromatic ethyl cinnamate groups connected to a benzene ring *via* an amide bond (Fig. 1). For the self-assembly of BTAC, we propose that both π–π interactions from aromatic rings and hydrogen bonding from the amide groups play very important roles.^16^ Achiral BTAC molecules could form supramolecular gels in a DMF/H_2_O mixture (v/v: 5/2) with spontaneous symmetry breaking, as proved by the unequal amount of left- and right-handed twists and the strong circular dichroism (CD) signals.^16^ In this case, supramolecular chirality can be obtained from the gelation of achiral BTAC molecules. The handedness of these chiral assemblies can be left-handed or right-handed by chance. Moreover, when BTAC gels in a DMF/H_2_O mixture (v/v: 5/2) are irradiated with 330 nm light, strong fluorescence with an emission maximum at 448 nm is detected (Fig. 2b), even though BTAC molecules themselves have very weak fluorescence in DMF solution. The absolute fluorescence quantum yield (*Φ*_F_) of BTAC solution in DMF is 0.003, while the BTAC gels have very strong fluorescence with *Φ*_F_ equal to 0.105. This significant gelation-induced fluorescence enhancement encouraged us to investigate the CPL response of these supramolecular gels which consist of only achiral molecular building blocks.

Amazingly, from different batches of BTAC gels in DMF/H_2_O mixtures (v/v: 5/2), strong CPL signals with different handedness and an emission maximum at 427 nm can be observed (Fig. 2b and S1†), highlighting excited-state supramolecular chirality of these assemblies formed by achiral molecular building blocks. In contrast, no CPL signals can be detected from BTAC solution (Fig. S2†). For understanding the relationship between the ground-state supramolecular chirality and excited-state supramolecular chirality of BTAC gels, the correlation between the CD signs and CPL signs was studied. The results reveal that the samples with a positive Cotton effect display left-handed CPL, while the samples with a negative Cotton effect display right-handed CPL (Fig. 2).

The magnitude of the circular polarisation at the ground state is defined as *g*_CD_ = 2 × (*ε*_L_ – *ε*_R_)/(*ε*_L_ + *ε*_R_), where *ε*_L_ and *ε*_R_ refer to the extinction coefficients for left- and right-handed circularly polarised light, respectively. Experimentally, the value of *g*_CD_ is defined as *g*_CD_ = [ellipticity/32 980]/absorbance at the CD extremum. The magnitude of CPL can be evaluated by the luminescence dissymmetry factor (*g*_lum_), which is defined as *g*_lum_ = 2 × (*I*_L_ – *I*_R_)/(*I*_L_ + *I*_R_), where *I*_L_ and *I*_R_ refer to the intensity of left- and right-handed CPL, respectively. The maximum *g*_lum_ value ranges from +2 for an ideal left CPL to –2 for an ideal right CPL, while *g*_lum_ = 0 corresponds to no circular polarization of the luminescence. Experimentally, the CPL was measured using a JASCO CPL-200 spectrometer, and the value of *g*_lum_ is defined as *g*_lum_ = [ellipticity/(32 980/ln 10)]/total fluorescence intensity at the CPL extremum.

Although BTAC gels were formed by identical achiral molecules, the absolute value of the dissymmetry factor (|*g*_lum_|) of their CPL signals is about 0.80 × 10^–2^ (Fig. 2b), which is much larger than those |*g*_lum_| values of some chiral organic molecules in solution or in the solid state. To the best of our knowledge, this is the first example that a supramolecular gel, prepared by only small achiral molecules, exhibits CPL feature without any chiral additives.

### Stirring-induced CPL enhancement

It has been reported that vortex stirring would lead to symmetry breaking and produce supramolecular chirality for some supramolecular assemblies formed by achiral molecules.^17^ More importantly, the handedness of supramolecular chirality of some systems, such as porphyrin assembly in solution, can be controlled by changing the direction of stirring.^17^ In contrast, for BTAC gels, the change from achiral molecules to chiral supramolecular assemblies is established simply upon gelation, no external mechanical force is necessary. In this context, it is also interesting to evaluate the effect of vortex stirring on the chirality of BTAC assemblies. Can mechanical force manipulate the symmetry breaking from gelation and finally decide the handedness of supramolecular chirality?

**Fig. 3:**
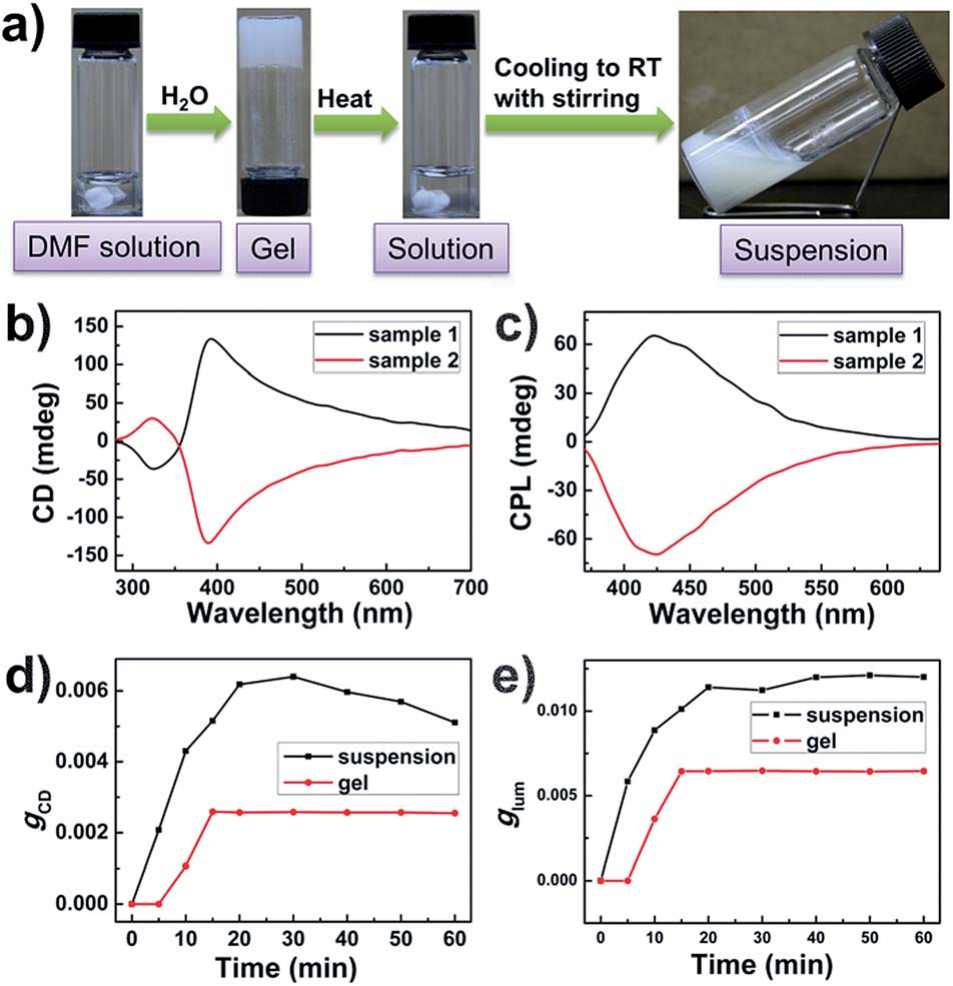
(a) Photographs showing BTAC assemblies (1% w/v) in DMF/H_2_O (v/v: 5/2) upon 900 rpm stirring during gelation; (b and c) CD spectra (b) and CPL spectra (c) of BTAC assemblies (1% w/v) after 900 rpm clockwise stirring during gelation; the sample with a negative Cotton effect displays right-handed CPL (red curve), while the sample with a positive Cotton effect shows left-handed CPL (black curve); (d and e) changes of *g*_CD_ (d) and *g*_lum_ (e) of BTAC gels (red curves) (1% w/v) and suspensions under 900 rpm stirring during gelation (black curves) in DMF/H_2_O (v/v: 5/2); suspensions under 900 rpm stirring during gelation show much larger dissymmetry factor than that of BTAC gels without stirring.

The BTAC gels formed in DMF/H_2_O (v/v: 5/2) are thermally reversible, and vortex stirring can be introduced into the system during the gelation process (Fig. 3a). The self-assembly of BTAC under stirring was performed in a 5 mL vial containing a 5.0 × 10.0 mm Teflon-coated magnetic stirring bar at the bottom, and clockwise (CW) or counterclockwise (CCW) stirring at 900 rpm was applied during the sol–gel process. The CD and CPL spectra of BTAC assemblies before and after vortex stirring with different directions were studied in detail.

**Fig. 4:**
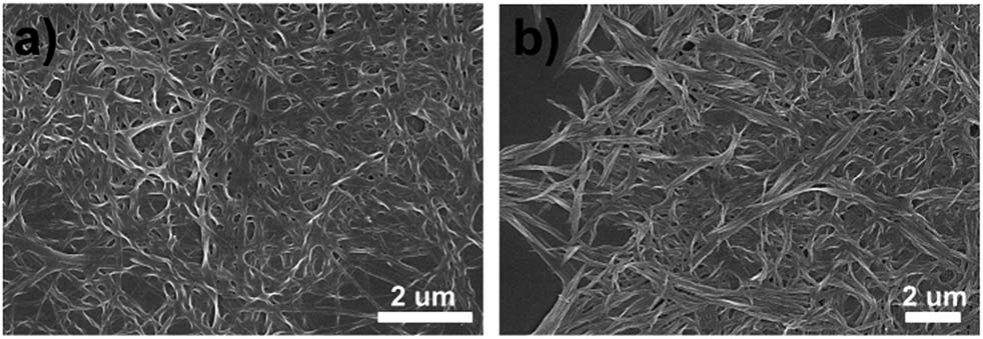
SEM images of BTAC gel (a) and the corresponding BTAC suspension after 900 rpm clockwise stirring during gelation (b) in DMF/H_2_O (v/v: 5/2).

Interestingly, although the gels are broken into suspensions upon long time stirring during gelation, the CD and CPL signals of BTAC assemblies could always be detected readily. The results show that BTAC suspensions show stronger CD and CPL signals after stirring (Fig. 3b and c). The strong CD signals with negligible linear dichroism (LD) artefacts are also confirmed by the LD spectrum (Fig. S3†). The SEM image of the BTAC assemblies after stirring also shows chiral twists, which are similar to the nanostructures of as-prepared BTAC gels (Fig. 4).

Most impressively, compared with the case of BTAC gels without stirring, both the CD signals and CPL activity of BTAC assemblies are found to be enhanced after stirring, as shown in Fig. 3 and S4.† Thus, if as-prepared BTAC gels have a positive Cotton effect and left-handed CPL, respectively, they will have a more intense positive Cotton effect and left-handed CPL after stirring, whether the stirring was clockwise (CW) or counterclockwise (CCW) (Fig. S5†). Thus, it is interesting to note that in our case, the handedness of the supramolecular chirality cannot be controlled by the stirring direction. Once the handedness has been decided by spontaneous symmetry breaking in the earlier stage of self-assembly, it cannot be changed by an external macroscopic mechanical force. The vortex stirring cannot change the interactions between different molecules. However, the stirring is able to enhance certain signals. Presumably it is because vortex stirring could promote the orderly arrangement of supramolecular assemblies around the nanoscale. This case can be proved by the scanning electron microscopy (SEM) images, in which many large assemblies can be detected after stirring (Fig. 4). Moreover, the unchanged molecular packing mode of BTAC assemblies before and after stirring can be demonstrated by the X-ray diffraction (XRD) patterns (Fig. S6†).

It is worth mentioning that stirring has to be applied during the sol–gel process to increase the supramolecular chirality of BTAC assemblies. Stirring after gelation cannot increase the intensity of the corresponding CD and CPL signals (Fig. S7†).

For further understanding the stirring-enhanced supramolecular chirality, we altered the stirring speeds during the self-assembly process, and the CD spectra of different samples after diverse stirring speeds were recorded. Interestingly, very slow stirring rates (300 rpm) could decrease the *g*-factor (Fig. S8†). Perhaps, slow stirring rates cannot promote the orderly arrangement of nanostructures. However, enhancement of the supramolecular chirality can be clearly detected when the stirring speed is further increased (Fig. S8†). Thus, 900 rpm stirring was selected for general investigation. The influence from the stirring time is also explored. Upon long time stirring, BTAC assemblies would become suspensions with good dispersion (Fig. S9†). The results show that both the CPL and CD signals come to their maximum values after about 30 minutes stirring at 900 rpm. However, further prolonging the stirring time does not improve the supramolecular chirality further (Fig. 3d and e). Compared with the change of CD intensity of BTAC gels without stirring (Fig. 3d), the enhancement of supramolecular chirality by mechanical force is remarkable. It is worth mentioning that a very high luminescence dissymmetry factor (*g*_lum_ = 1.2 × 10^–2^) is achieved upon long time stirring (Fig. 3e). Therefore, the CPL materials constructed by supramolecular gels have very nice tunability. Altogether, CPL supramolecular gels are fabricated by simple achiral *C*_3_-symmetric molecules, and the intensity of the corresponding CPL signals can be further enhanced *via* mechanical stirring.

### Chiral amine-induced enhancement and control of CPL

It has been reported that the chirality of supramolecular assemblies can be controlled by chiral additives.^18^ We have found that chiral organic amines have the capability to pass on their molecular chirality to the supramolecular assemblies formed by achiral BTAC molecules and therefore control the handedness of these chiral assemblies.^16^

**Fig. 5:**
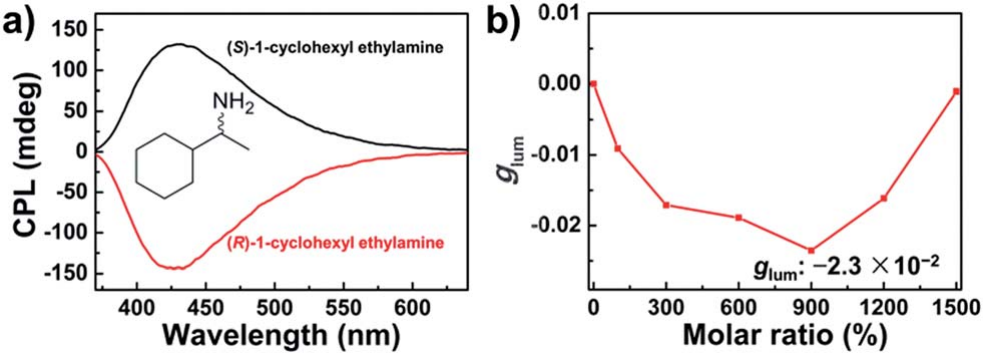
(a) CPL spectra of BTAC gels (1% w/v) containing 900 mol% chiral 1-cyclohexyl ethylamine in DMF/H_2_O (v/v: 5/2); (b) the dissymmetry factors (*g*_lum_) of BTAC gels with different molar ratios of (*R*)-1-cyclohexyl ethylamine/BTAC.

With regard to the supramolecular chirality of BTAC assemblies on the excited-state optical activity, addition of the chiral amines not only controls the handedness of CPL but also enhances the amplitude of CPL. For the BTAC gels containing (*R*)-1-cyclohexyl ethylamine with a molar ratio of BTAC/amine = 1 : 9, a strong right-handed CPL signal was detected (Fig. 5a). In contrast, in the case of BTAC gels containing (*S*)-1-cyclohexyl ethylamine, the mirror-image CPL spectrum was discerned (Fig. 5a). Remarkably, the dissymmetry factor (*g*_lum_) increases greatly to ±2.3 × 10^–2^ with doping chiral organic amines. Therefore, both the intensity and handedness of CPL signals can be easily modulated. On the other hand, although a higher concentration of the chiral amine in the system could result in higher CPL activity, overloaded chiral components could also destroy the assembly and decrease the intensity of CPL peaks (Fig. 5b).

## Conclusions

In conclusion, the supramolecular gels with strong CPL responses have been fabricated from a very simple achiral *C*_3_-symmetric gelator. Moreover, the excited-state supramolecular chirality of these materials can be controlled perfectly. Thus, the luminescence dissymmetry factor (*g*_lum_) can be enhanced greatly by simply using mechanical stirring or adding chiral amine dopants, which is also useful for controlling the handedness of CPL signals. Considering the explosive growth of demand for developing novel CPL materials, our work opens new strategies to achieve low-cost CPL supramolecular gels, which are easily fabricated into different devices with desirable flexibility and biocompatibility. Most of all, our results also firstly demonstrate that excited-state supramolecular chirality can also be obtained from the self-assembly of identical small achiral molecules.

## Experimental section

### Instruments and methods

Scanning electron microscopy (SEM) was performed on a Hitachi S-4800 FE-SEM with an accelerating voltage of 10 kV. Before SEM measurements, the samples on silicon wafers were coated with a thin layer of Pt to increase the contrast. UV-Vis, CD and LD spectra were obtained using JASCO UV-550 and JASCO J-810 spectrometers, respectively. CPL measurements were performed with a JASCO CPL-200 spectrometer. 0.1 mm cuvettes were used for measuring the UV-Vis, CD, LD and CPL spectra of samples. For the measurement of CD spectra, the cuvette was placed perpendicularly to the light path of the CD spectrometer and rotated within the cuvette plane, in order to rule out the possibility of birefringence phenomena and eliminate the possible angle dependence of the CD signals. Fluorescence spectra were recorded on a Hitachi F-4500 fluorescence spectrophotometer. The absolute fluorescence quantum yield was measured by using an absolute PL quantum yield spectrometer (Hamamatsu Photonics) with a calibrated integrating sphere. X-ray diffraction (XRD) analysis was performed on a Rigaku D/Max-2500 X-ray diffractometer (Japan) with CuKα radiation (*λ* = 1.5406 Å), which was operated at a voltage of 40 kV and a current of 200 mA.

### Materials

All the starting materials and solvents were obtained from commercial suppliers and used as received. 1,3,5-Benzenetricarbonyl trichloride and ethyl 4-aminocinnamate were purchased from Alfa Aesar. The *C*_3_-symmetric gelator (BTAC) was synthesized by following the method reported previously.^16^ Milli-Q water (18.2 MΩ cm) was used in all cases. (*R*)-1-Cyclohexyl ethylamine and (*S*)-1-cyclohexyl ethylamine were purchased from TCI and used as received.
