# Supplementary material for: Strong circularly polarized luminescence from the supramolecular gels of an achiral gelator: tunable intensity and handedness
Source: Chem Sci. 2015 Apr 30;6(7):4267–72. doi: 10.1039/c5sc01056j (PMC5707475; doi:10.1039/c5sc01056j)
Supplement: Supplementary file 1 [file SC-006-C5SC01056J-s001.pdf]

Electronic Supplementary Material (ESI) for Chemical Science.
This journal is © The Royal Society of Chemistry 2015

Electronic Supplementary Information

Strong Circularly Polarized Luminescence from the Supramolecular Gels of an Achiral Gelator: Tunable Intensity and Handedness

Zhaocun Shen,[a] Tianyu Wang,*[a] Lin Shi,[b] Zhiyong Tang,[b] and Minghua Liu*[ab]

[a] Beijing National Laboratory for Molecular Science, CAS Key Laboratory of Colloid, Interface and Chemical Thermodynamics, Institute of Chemistry, Chinese Academy of Sciences, Beijing 100190, P. R. China.

[b] Laboratory of Nanomaterials, National Center for Nanoscience and Technology, Beijing 100190, P. R. China.

Supplementary figures

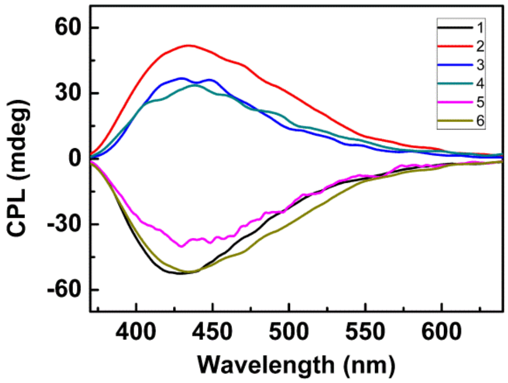

Fig. S1 CPL spectra of BTAC gels (1% wt/vol) in DMF/H_2O (v/v: 5/2) from different batches excited at 330 nm.

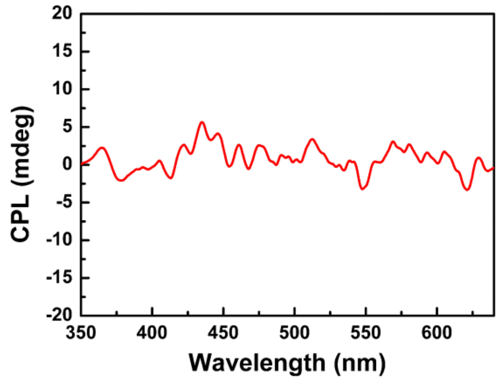

Fig. S2 CPL spectrum of BTAC hot solution (1% wt/vol) in DMF/H_2O (v/v: 5/2) excited at 330nm.

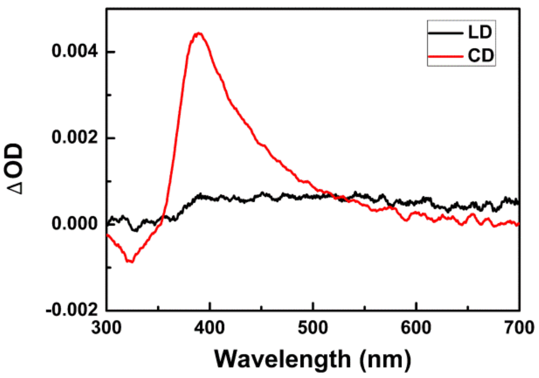

Fig. S3 The typical CD and LD spectra of BTAC assemblies (1% wt/vol) obtained after 900 rpm stirring during gelation, which are unified as the same unit (Δ OD).

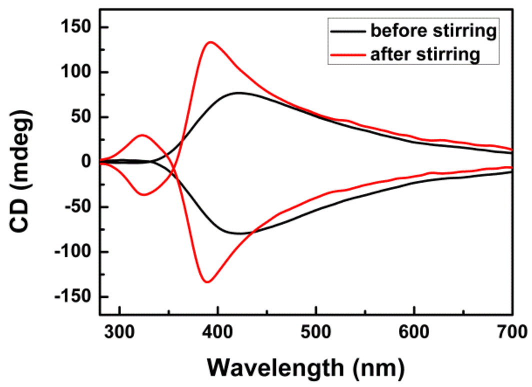

Fig. S4 CD spectra of BTAC assemblies (1% wt/vol) in DMF/H_2O (v/v: 5/2) before stiring (black curves)and after 900 rpm clockwise stirring during gelation (red curves).

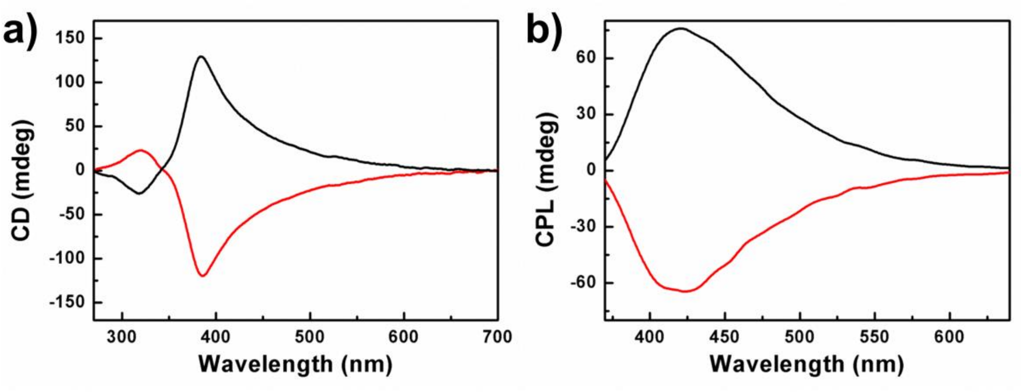

Fig. S5 CD spectra (a) and CPL spectra (b) of BTAC assemblies (1% wt/vol) in DMF/H_2O (v/v: 5/2) after 900 rpm counterclockwise stirring during gelation.

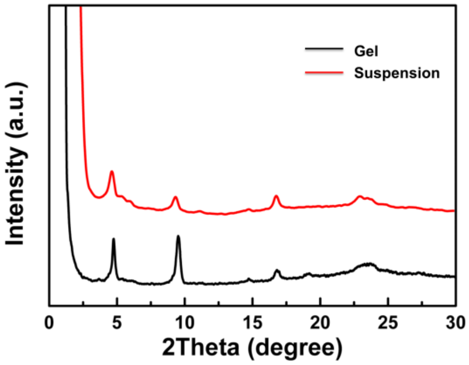

Fig. S6 XRD patterns of BTAC gels (black curves) and the corresponding BTAC suspensions after 900 rpm clockwise stirring during gelation (red curves) in DMF/H_2O (v/v: 5/2).

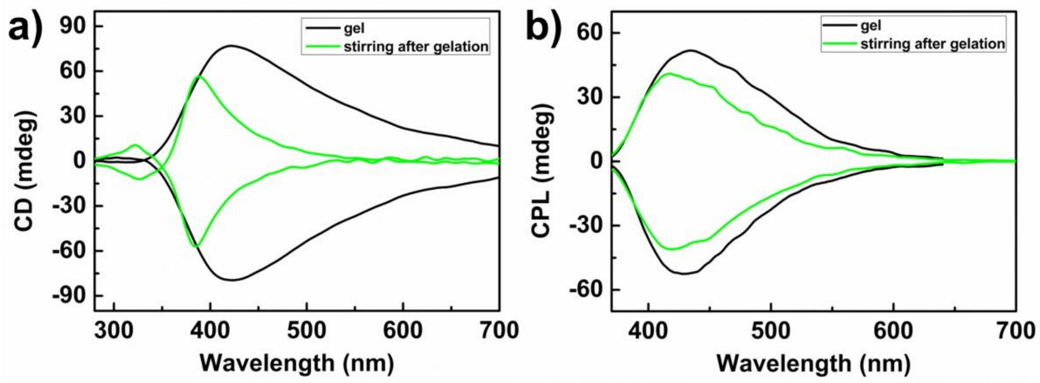

Fig. S7 CD spectra (a) and CPL spectra (b) of BTAC gels (black curves) and suspensions obtained by 900 rpm clockwise stirring after gelation (green curves) in DMF/H_2O (v/v: 5/2).

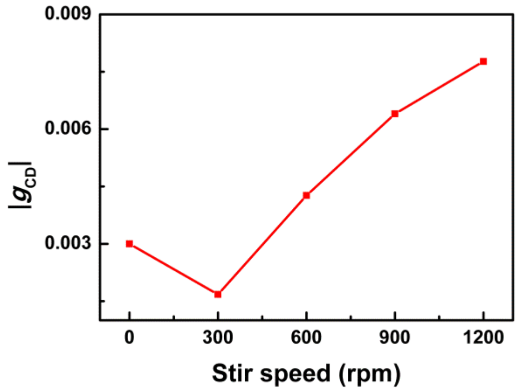

Fig. S8 The absolute value $|g_{CD}|$ of BTAC assemblies (1% wt/vol) in DMF/H_2O (v/v: 5/2) under diverse stirring speeds.

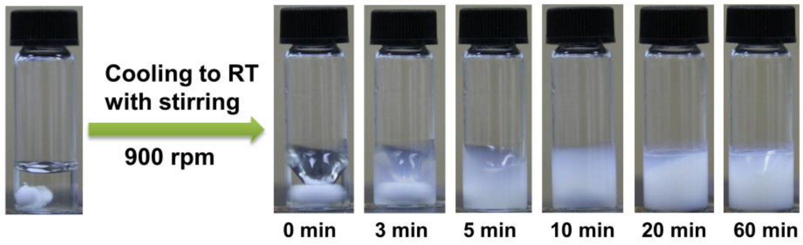

Fig. S9 Appearance change with stirring time when the hot solution (1% wt/vol) is cooled down to room temperature with stirring at 900 rpm.
